# Adolescents’ Perceptions and Experiences of Their Responsibilities for Their Alcohol Use—A Group Interview Study

**DOI:** 10.3390/children8030214

**Published:** 2021-03-11

**Authors:** Mari A. Mynttinen, Kaisa E. Mishina, Mari K. Kangasniemi

**Affiliations:** 1 Department of Nursing Science, Faculty of Medicine, University of Turku, 20014 Turku, Finland; mari.mynttinen@utu.fi (M.A.M.); mari.kangasniemi@utu.fi (M.K.K.); 2 Department of Child Psychiatry, Faculty of Medicine, University of Turku, 20014 Turku, Finland; 3 INVEST Research Flagship Center, University of Turku, 20014 Turku, Finland

**Keywords:** adolescents, alcohol use, group interviews, qualitative study, responsibilities

## Abstract

Young people often experiment with alcohol during adolescence, which is a period of their life that is characterized by increasing responsibility. Knowing how adolescents perceive responsibilities with regard to their alcohol use could prevent their alcohol consumption and help them to take responsibility for this aspect of their lives. This study describes adolescents’ perceptions and experiences of their responsibilities for alcohol use. We used a qualitative descriptive method that focused on 87 adolescents aged 14–16 years, from two schools. They took part in semi-structured interviews in 19 groups in Finland in 2017. The data were analyzed using inductive content analysis. The adolescents described alcohol as harmful, but tempting, and said that they were developing a sense of responsibility for their alcohol use. They were responsible for their own wellbeing, behaving responsibly if they drank and intervening in peers’ alcohol use. They talked about how their parents had unquestionable responsibilities to care about whether adolescents drank alcohol. Their parents’ responsibilities related to the guidance they gave, how strict they were and how they responded to adolescents using alcohol. Anonymous and intense support from authorities encouraged adolescents to learn to take responsibility. Identifying and focusing on their responsibilities could help adolescents to develop into healthy individuals and increase their awareness of the need to avoid alcohol. Parents may also need support to meet their responsibilities.

## 1. Introduction

Alcohol is still the most common psychoactive substance used by adolescents worldwide, although its consumption has steadily declined in many countries [1,2,3]. The World Health Organization (WHO) defines adolescents as individuals aged 10–19 [4] and has estimated that 50–70% have tried alcohol by the time they reach the age of 15. More than a quarter (27%) of adolescents aged 15–19 years drink alcohol, which represents a global figure of 155 million. European adolescents have the highest prevalence rates for alcohol use in that age group [3]. A study carried out in Finland found that just over a fifth (22%) of adolescents aged 15–16 had been drunk during the last month and that 11% of boys and 3% of girls felt that there were no health risks associated with regular alcohol use. The adolescents also said that getting alcohol was easy, especially from friends [1]. It is very clear that the short-term and long-term consequences of alcohol use are harmful for adolescents’ health and futures [5,6,7] and this is a major concern for families and those working in preventive health care [8,9].

Adolescents’ perceptions and experiences of alcohol vary and studies have found that those who perceived alcohol use as less risky often drink more [10,11]. Adolescents even saw alcohol use as an entirely normal and acceptable part of their social life, as long as it was kept under control [12]. Adolescents are often aware of the negative consequences of alcohol use, such as death, but they may think that bad things only happen to other people. For example, one study reported that adolescents felt untouchable and totally in control [13]. Other studies reported that adolescents used alcohol to show their friends that they were friendly, sociable, fun and had something in common with them [12,14].

Research has found that adolescents used alcohol to control their feelings, such as belonging to a group [15], pleasure, freedom [13] and relaxation [14]. They also used it to cope with feelings of depression, unhappiness and boredom and got drunk to boost their confidence [14,16]. It is also worth noting that adolescents might not make a conscious decision to drink alcohol. They may use it because they are influenced by their socioeconomic background, such as their social and family environment and education [11,13]. In addition, the way that they regulate their behavior is still developing during adolescence and their ability to make healthy choices improves as their cognitive abilities evolve [17].

As well as being a time when individuals like to experiment with alcohol, adolescence is a period of increasing responsibility [18]. Responsibility has been defined as how someone controls their own choices and personal affairs [19] and how their morals are perceived by others [20]. People are seen as responsible if they are doing what they are required to do and can justify their actions. This requires knowledge, freedom and the power to act purposefully [21]. One study reported that adolescents said that their responsibility for their health meant that they needed to accomplish the practical everyday tasks required of them, such as taking care of their health and their peers’ health [22]. This means that adolescents’ perceptions of their responsibilities have an effect on their use of alcohol. Their perceptions that alcohol provides their lives with meaning, such as their need for identity and hedonic pleasures, often lead adolescents to knowingly make unhealthy decisions and choices, despite the risks [15].

Previous research has focused on responsibilities for adolescents’ alcohol use from their own developmental perspective [23] and from their parents’ point of view [24]. Research has mainly focused on adolescents’ alcohol consumption [25] and its consequences [5,6]. It is important that adolescents protect their future health by learning to make their own responsible healthy decisions and choices [22]. Support from families, schools and peers has been found to prevent, and reduce, adolescents’ drinking [26,27]. However, we need to know more about adolescents’ own perspectives of their responsibilities with regard to their alcohol use and what support adolescents feel they need [24,28]. The Finnish Alcohol Act (1102/2017) states that people under 18 years of age are not allowed to buy or consume alcohol [29]. Since previous research on this topic has been scarce, the aim of this study was to describe adolescents’ perceptions and experiences of responsibilities with regard to their alcohol use. The knowledge produced by this study will help us to understand adolescents’ perspectives and how to support them to take responsibility when it comes to alcohol. This will enable policy makers and healthcare services to develop preventative measures, and target resources that prevent adolescents’ alcohol use and support their growth, development and future health.

## 2. Materials and Methods

This was a cross-sectional, phenomenological, qualitative study that used semi-structured group interviews. The consolidated criteria for reporting qualitative research (COREQ) was used to ensure explicit and comprehensive reporting [30].

We used purposive sampling to collect data from two public secondary schools in Eastern Finland in 2017. Purposively selecting one rural and one urban school ensured the heterogeneity of the findings. There were 416 ninth-grade students, aged 14–16, in the two schools: 282 in the urban school and 134 in the rural school. After we obtained approval from the Committee on Research Ethics of the University of Eastern Finland and permission from the school districts, the first author (M.M) contacted the head teachers for their permission to recruit participants. The recruitment process had two stages. First, the head teachers emailed letters about the study to their ninth-grade students and their parents. They were given two weeks to decide whether they wanted to participate. After the two weeks has elapsed, the researcher visited the schools, introduced herself to the adolescents, presented the study aims and invited them to take part. The adolescents were given information letters about the study to take home and the teachers passed this on to any students who were absent from school on the day of the visit. Seven adolescents aged 14 years old returned their parents’ signed consent forms to the researcher, as they were below the age of consent. Those aged 15 and 16 were able to provide their own consent under Finnish law, according to the ethical principles of research in Finland [31] and Finnish law [32], and 80 did so. The overall response rate was 21%. The focus group interviews were carried out once informed consent was received.

The inclusion criteria was that adolescents had to be in the ninth grade in one of the two secondary schools, which typically covered Finnish students aged 14–16 years. The 87 adolescents (50 girls and 37 boys) had 2–12 family members, including step families. The majority, 54 students, had 4–5 family members (Table 1). Most of the participants (87%) were from the urban school. After the adolescents were enrolled in the study, the researcher and teachers worked with them to divide them into a number of interview groups. The adolescents were able to choose what interview group they were in, as it was important that they felt comfortable talking in front of the other members. This resulted in the 87 adolescents being placed in 19 groups: eight for just girls, six for just boys and five mixed groups. The groups ranged from three to six participants.

We chose semi-structured group interviews because we found this was the best way to encourage the adolescents to share their perceptions and experiences on the shared topic of responsibilities for alcohol use [33]. This method was also selected because its socially oriented nature reflects real life, where participants influence, and are influenced, by others [34]. The study used a semi-structured interview guide that was based on previous knowledge and covered the predefined interview themes [35]. The interview guide consisted of three stages: setting the scene, exploring the key theme of the study and ending the interview and asking closing questions. There were four interview themes. First, we asked the adolescents what they thought about them and their peers using alcohol and how they used it. Second, we explored how they saw their parents’ roles and what involvement they had in whether they drank alcohol. The third theme was what the adolescents felt their responsibilities were with regard to using alcohol. The final theme was what kind of support would prevent them from using alcohol and help them with the problems that using alcohol could cause among adolescents. The adolescents were not asked about their alcohol consumption, but they could share that information if they wanted to. Each interview theme included three to six predesigned follow-up questions, which were designed to clarify the adolescents’ responses and obtain further information [35,36]. For example, we asked the adolescents to provide examples to illustrate their comments and asked them how they felt about something they had said.

The researcher (M.M.) carried out the audiotaped group interviews in quiet classrooms during the school day and no other people were present. At the beginning of each interview, the researcher introduced the research, and herself, to the participants. The researcher encouraged the participants to talk freely about the topic and only interrupted them when they said something that needed elaboration or clarification. If necessary, the researcher rephrased and repeated the questions [37]. Field notes were made during the interview. At the end of the group interviews, the researcher asked the participants if they wanted to add something. The audiotaped interviews lasted from 35 min to 2 h 18 min and the average length was 1 h and 7 min. We achieved data saturation after the fifteenth interview, but we continued with the other four interviews to ensure that no new content emerged [38]. The total length of the 19 interviews was 21 h and 19 min.

We used the inductive content analysis method [39] for the data analysis. First the data were manually transcribed verbatim from the audio recordings by the researcher (M.M). This generated 10,465 lines of text, which equated to 647 A4 transcribed Microsoft Word pages with 1.5 line spacing. We analyzed the data manually. First the researcher (M.M) read the text thoroughly several times and extracted the expressions, namely selected words, sentences or entire paragraphs that related to the same central meaning units (Table 2). After the expressions had been condensed, the researcher (M.M) combined them into single and grouped codes based on their similarities and differences. After the codes were grouped, the research group continued the analysis process together. The research team held face-to-face meetings to discuss the data grouping, to make sure that the data capture was complete. The group also abstracted and named the condensed groups inductively, to provide descriptive subcategories and categories [39]. These shared discussions identified 33 subcategories and 10 categories derived from the data. The research team then placed the data into the four main categories that reflected the four interview themes described above. Then, the first author wrote up the results.

The study received ethical approval from the Committee on Research Ethics of the University of Eastern Finland (Statement UEF/12/2017), permission from the participating schools and written, informed consent from the adolescents or their families, depending on the students’ ages. The interviews were carried out by the researcher (M.M), who is a female public health nurse, has a Master’s Degree in health sciences and has worked in school and student health services. There were no prior relationships between the researcher and participants. At the beginning of each interview, the researcher emphasized the confidential nature of the interview and stressed that anything they heard during the focus group should not be discussed elsewhere. The participants agreed to respect the need for confidentiality. We agreed that if any harmful behavior emerged during the interviews that could jeopardize the safety of the participant and/or other adolescents, the researcher would inform the authorities about this. However, this was not necessary.

## 3. Results

The adolescents’ perceptions and experiences of responsibilities in relation to using alcohol focused on four key areas. These were: (i) alcohol use was harmful but tempting, (ii) adolescents had a developing sense of responsibility for alcohol use, (iii) parents were unquestionably responsible for adolescents’ alcohol use and (iv) support provided by authorities needed to focus on certain important characteristics (Table 3).

### 3.1. Alcohol Use Was Harmful but Tempting

Adolescents felt that it was their responsibility to understand that using alcohol was senseless, because it was harmful. However, some of them said that they realized that drinking might be tempting for some other adolescents.

#### 3.1.1. Senseless Use of Alcohol

The participants did not see any sense, interest or benefits in alcohol use. Instead they wanted to enjoy good health without alcohol, now and in the future. The adolescents emphasized that alcohol was a harmful and dangerous substance that had damaging consequences for health, especially for the liver and brain. It retarded their physical growth and could affect their mental health, for example by causing depression. Some adolescents mentioned that the gateway drug effect was a terrifying possibility. They felt that using weaker alcoholic drinks would increase their use of stronger alcohol or other drugs. Some of them had also noticed that when their peers drank they had an impaired appearance and displayed changes in behavior, such as a lack of interest in physical activities. Based on their own bad experiences, or things they had heard, some adolescents were worried that using alcohol could involve them in traffic accidents that could kill them.

“I have not seen a fit and healthy substance user yet. Alcohol has many effects on health.” (Group 17).

“Alcohol use has a noticeable effect on someone’s appearance.” (Group 1).

“If you begin to drink when you are very young, you will probably drink for the rest of your life and you will not live a long life if you end up on the wrong path.” (Group 19).

“Someone could have choked on their own vomit or frozen to death in winter.” (Group 19).

Adolescents did not want to endanger their future by excessive alcohol use. They were afraid that excessive drinking could stop them getting into their desired school, receiving a good education and getting a job. This was because adolescents felt that if they did something senseless when they were drunk, the authorities, such as the police and social workers, would make a note of it and they could end up with a criminal record. They felt that drinking would spoil their image and they wanted to protect their reputation. Adolescents said that drinking did not make sense, as it often complicated their relationships with their parents by causing problems and arguments. When they did not drink, their parents valued them and considered them trustworthy. They did not want to betray their parents’ trust and spoil their relationships with them by drinking.

“Rumors spread quickly. For example, if you have been embarrassing yourself by hanging around naked in the city center, you will get a pretty bad reputation.” (Group 15) “It feels really good when your parents trust you. They know that you will not become a heavy drinker.” (Group 1).

“If I started drinking, I would lose my parents’ confidence in me. It is very important for me what my parents and family think of me, because we are so close.” (Group 1).

Adolescents pointed out that excessive alcohol use caused additional expenses for society by increasing national healthcare costs. Some of them said the number of highly educated people would decrease in their country due to alcohol use. Adolescents did not want to become outcasts and fall by the wayside, like some people “running around the social services center” did. Some of them felt that people who drank often assumed that they would get money from social services to enable them to drink further. At an individual level, some adolescents said that alcohol use was not wise, because they could lose their pocket money. It was their responsibility to be aware of these consequences.

“Society is responsible for the cost of treating diseases caused by alcohol.” (Group 2).

“There would not be any engineers graduating from Finnish universities, nor any other highly educated people, if they abused alcohol.” (Group 16).

“You could end up standing in line at the social welfare offices asking to get more money, so that you could buy more and more beer.” (Group 4).

#### 3.1.2. Desired Benefits of Alcohol Use

Although the adolescents said that using alcohol was harmful, they could see that it was tempting for some of their peers. Adolescents felt that their peers often used alcohol because they felt the benefits were desirable, including feeling like they belonged to a group, gaining approval and attention from their peers and having fun. Some of the participants felt this was understandable, because no one wanted to be alone or feel left out. However, some of the girls felt that they would have had even more fun if they were sober. Some adolescents knew that peers might use alcohol to regulate and control their feelings, to help them solve problems and provide an escape from difficult emotions. Adolescents thought that someone might deliberately want to hurt themselves by using alcohol to relieve stress or to relax. They said that adolescents were curious to taste alcohol, because they felt it might offer new experiences and bring excitement to their mundane lives.

“Certainly, the use of alcohol can increase the feelings of belonging to a group.” (Group 2).

“You will become an outsider if you do not go drinking with your peers.” (Group 8).

“Sometimes people can drink to get drunk. They want to be in a different place to when they are sober.” (Group 3).

“You might drink to escape everyday life.” (Group 17).

“You get a feeling of excitement when you drink alcohol, a kind of adrenaline.” (Group 5).

“Yes, alcohol tastes nice and it is good to try something new.” (Group 13).

### 3.2. Adolescents Developing a Sense of Responsibility

Most of the adolescents felt that they were capable of developing a sense of their responsibilities with regards to alcohol use. Adolescents’ perceptions and experiences of the skills they needed to take responsibility for alcohol related to taking care of their own wellbeing, their views on responsible behavior when drinking and intervening when they were concerned about their peers’ alcohol use.

#### 3.2.1. Taking Care of Their Own Wellbeing

The participants felt that adolescents in their age group frequently wanted to be responsible for taking care of themselves and their own wellbeing and were learning to do this. This meant that they needed the ability and knowledge to make the right decisions and take responsibility for their drinking. Adolescents felt that underage drinking was unquestionably wrong and did not belong in their life. This view was based on the law, which bans underage drinking in order to protect children’s health, wellbeing and development.

“It is basically our responsibility to take care of ourselves.” (Group 6).

“You make the decision yourself whether you accept a drink or not when someone offers you one.” (Group 7).

“At our age, we know what we are doing, what is right and what is wrong, what we can and cannot do. It is no longer a matter of debate.” (Group 4).

“Children are not supposed to drink. It is prohibited for children under 18 years of age.” (Group 14).

“And it is a crime anyway.” (Group 16).

Adolescents felt that they would benefit from talking about their problems when they were developing their sense of responsibility. They said that it was responsible behavior, and a necessary part of taking care of their own wellbeing, to talk to friends or adults about concerns and difficulties with alcohol use. Although talking about this issue could sometimes be difficult, they felt they were learning to do it. Some adolescents felt it was easier to open up to friends, because they felt equal and had more confidence. Friends were often the first ones they turned to with problems and to ask for help, even before their own parents. Adolescents said that friends encouraged them and did not shout or punish them. They also emphasized that taking care of their own wellbeing required the ability to look for support and help when necessary and to receive that help with an open mind. According to some adolescents, the use of alcohol could increase if they did not consider the need for help and support seriously enough. They said that they could refuse help because they did not recognize that they needed support. Adolescents felt that having help forced on them would be useless.

“You can talk to friends who you trust. You know they will not talk about it to anyone else.” (Group 4).

“You should have a positive and responsive attitude to help and support, instead of ignoring your need for help. You should not be like this does not help, this is totally rubbish.” (Group 9).

“If a person is involuntarily forced into an institution, they might want to increase their use of alcohol, just because they want to be rebellious.” (Group 16).

#### 3.2.2. Responsible Behavior When Drinking

Adolescents discussed the skills they needed to behave responsibly when they were drinking and saw these as part of their developing sense of responsibility. Some of the adolescents said the only responsible behavior was not to use alcohol. They described themselves as being abstinent or having occasionally tasted a little diluted alcohol. Most of the adolescents had used alcohol in moderation, were well behaved and considered this to be responsible. They did not binge drink, but described their alcohol use as carefully considered and self-controlled. In order to be responsible, they made sure that their parents were able to phone them when they were out with their friends. Some adolescents said that it was responsible to make sure they stayed safe by drinking in safe company, such as parents and/or friends, rather than with strangers. Some of them said that it was responsible to change their circle of friends from drinkers to non-drinkers if necessary. The adolescents talked about safe people to drink with.

“It would be better to drink with an adult who is sober. A family friend, parents or siblings, who could look after you, rather than going somewhere to drink alone, with some random drunk or with a bunch of strangers.” (Group 4).

#### 3.2.3. Intervening in Peers’ Alcohol Use

The adolescents felt that developing a sense of responsibility included intervening in their peers’ alcohol use. Some of them felt they already had the skills to do this. Intervening meant that adolescents made an effort to prevent peers drinking alcohol by providing sensible advice and restrictions. Some adolescents felt that taking care of peers included refusing to purchase or offer alcohol to them. One participant said that her mother encouraged her to take care of her friends and that she did this. Being responsible also involved helping peers to stop prolonged drinking and not leaving a drunk friend anywhere alone. If adolescents saw friends or others, especially small children, drunk they said it would be the responsible thing to do to contact the police or the parents of those who had been drinking. However, some adolescents wanted to protect their friends from getting into trouble.

“You can save your friend’s life by talking sense to them about the consequences if they drink every weekend.” (Group 4).

“You cannot just leave your friend somewhere on the side of the road. You must help. Anything can happen and you must take them home.” (Group 18).

“I dragged my drunk friend all the way through the port. There were police on every corner, so we had to go through narrow roads in order to get a taxi home without getting caught. Otherwise, I know he would have got into big trouble.” (Group 5).

Although most adolescents pointed out that it was their responsibility to intervene in their peers’ alcohol use, some of them felt it was not their responsibility to take care of drunk peers. They felt that the responsibility lay with the person who was drinking and it would not be fair to sober friends. Some boys did not know how to help peers, even though they wanted to. They said that they would not know what to do if something happened to their drunk friends.

“At parties there are often one or two sober guys and it is their responsibility to take care of you when you are drunk. This is not fair on them. It is not their job really and it does not make any sense. If you drink, it is your responsibility to take care of yourself. It is not anyone else’s duty.” (Group 12).

### 3.3. Parents’ Unquestionable Responsibility for Adolescents’ Alcohol Use

Although adolescents were developing their own sense of responsibility, they discussed parents’ unquestionable responsibilities when it came to adolescents using alcohol. They emphasized that parents were adolescents’ primary caretakers and ultimately responsible for protecting them and stopping them using alcohol. It was the parents’ responsibility to guide adolescents towards responsible decision making and to consider how permissive they should be about alcohol use. Parents’ responsibilities also covered how they responded to their adolescents experimenting with alcohol.

#### 3.3.1. Guidance about Responsible Decision-Making

The adolescents said it was the parents’ responsibility to guide their children towards making independent, but responsible, decisions about whether to drink or not. This meant sharing their knowledge and advising them about the physical, mental and social consequences of alcohol use. Teaching sound judgment about decision-making meant that parents were clear about what was right and wrong for their children. Some boys thought that the parents of binge drinking adolescents had not raised them in the right way and, therefore, the adolescents’ drinking was the parents’ fault.

“It is not the parents’ job to tell children that they are not allowed to drink alcohol. Their task is to teach them the skills, and to give them advice, so they can make a judgement and be capable of making their own considered decisions.” (Group 12).

“Some adolescents do not control themselves at all. They do what they want and do not think. They just hang around and drink. Those are the ones who are badly raised.” (Group 13).

#### 3.3.2. Permissiveness and Alcohol Use

Participants said permissiveness by parents varied from total prohibition to indulgent alcohol use and this could be different between parents in the same family. For example, one girl said that her mother did not allow her to taste any alcohol, but her father did. Some of the adolescents said that their parents’ responsibilities were clear and they expected total prohibition. Adolescents said they had been punished by their parents when they were caught drinking. Punishments included taking their phones away, stopping them meeting their drinking friends or setting tight curfews. Some had been grounded, forbidden to play with computers, given long lectures on drinking or had their pocket money withheld. One boy said that he would be beaten if he was ever caught drinking. Even though adolescents understood the punishments were because their parents cared and felt responsible for them, they felt that frightening, blaming and even intimidating them was not effective. Some adolescents said that total prohibition, very strict rules and punishment often led to undesirable drinking, because they felt defiant.

“If I was punished, I would deliberately drink again. I would certainly disobey.” (Group 4).

“If parents prohibit it completely, it causes a kind of defiance among adolescents. It is a natural reaction, but unfortunately many parents do not realize this. I understand that they have had lots of problems with their children, but… it does not justify prohibiting everything.” (Group 12).

Some adolescents said their parents had given them permission to taste alcohol under their supervision, such as during toasts at a celebration like a wedding or birthday. However, adolescents were only allowed half a glass or one glass, which they felt was responsible. Some adolescents thought the law allowed them to drink under their parents’ supervision, but this is not the case in Finland.

“When you are young, you just do not have enough knowledge and you might act very irresponsibly. Adolescents must be taught the rules so that they can learn.” (Group 3).

“During a celebration, my grandparents might pour some wine for me, but not more than one glass. However, this does not mean that we are going to get sloshed together.” (Group 4).

“Giving a little alcohol to your own child under supervision is allowed isn’t it?” (Group 13).

“The law does not forbid tasting alcohol if a sober parent is supervising.” (Group 13).

According to the adolescents, some parents were not responsible and allowed them to drink alcohol without supervision. Some participants felt it would be weird, nonsensical and bad parenting if parents allowed the child to do what they wanted and even supplied them with alcohol. However, one boy disagreed and pointed out that giving them the freedom to drink could decrease alcohol use, as not all adults drank, even though they could.

“If a parent allows a child to drink and hang around sozzled until 5am, without letting their parents know where they are… this means they are bad parents and do not deserve to be a parent.” (Group 15).

“It could be better to allow them to drink, because a child might only drink occasionally because they are allowed to do so. A child might use this freedom to drink responsibly.” (Group 1).

#### 3.3.3. Parents’ Responses to Adolescents Experimenting with Alcohol

Adolescents said that parents’ responsibilities also covered how they responded to them experimenting with alcohol. They felt that parents should supervise what their children were doing, appropriately and actively, and should know where they were every day. Some adolescents said that most of the parents performed this task quite well and both the children and parents understood the rules. However, some parents could be negligent and even be unaware of their children’s whereabouts. Adolescents said that the worst thing an adult could do was abandon their own child and leave them to their own devices. They felt that this type of parental attitude could cause adolescents to drink, but they might also drink more if parents were overprotective about them experimenting with alcohol. They had heard that some parents had contacted social care workers, or even called the police, and felt that was an unusual and over-the-top reaction.

“The parents should definitely intervene. Letting your child hang out in the city every single weekend is not responsible parenting.” (Group 10).

“It is bad that some parents are not interested in their children.” (Group 11).

“As a parent, you do not leave or abandon your child.” (Group 10).

“Some parents are so strict that, if their child came home drunk, they would call the police.” (Group 18).

### 3.4. Important Characteristics of Support Sought from Authorities

In addition to the adolescents’ perceptions and experiences of their developing sense of responsibility, and the parents’ unquestionable responsibilities, adolescents felt that the authorities were also responsible for adolescents’ alcohol use. These authorities included social and healthcare workers, such as school nurses, social welfare officers, youth workers, teachers and the police. Their responsibilities included providing services like preventive counseling and, if needed, helping adolescents with problems they had as a result of alcohol use. The support that adolescents sought from authorities needed to guarantee certain characteristics.

#### 3.4.1. Anonymity

Adolescents thought that if they needed treatment or support for problems related to alcohol they should be able to access these anonymously. They were aware they could get confidential counseling and support from school personnel, such as the school nurse and school social worker, but they preferred to get help anonymously via the Internet, social media or by phone. When they called an anonymous helpline for adolescents, or shared their thoughts in a discussion forum on the Internet, they could receive support from a sympathetic person they did not know. That person was capable of understanding adolescents, identifying with them and did not judge them. Some adolescents felt that they would trust the authorities more if they made their obligation to confidentiality more visible.

“A stranger does not judge you when you call anonymously about your worries. I can imagine that accessing support and talking to someone would be easiest over the phone.” (Group 12).

“It should be more strongly emphasized that the authorities are specifically there to take care of things anonymously. You can tell them everything”. (Group 18).

#### 3.4.2. Detailed Insight and Knowledge

Adolescents pointed out that any support that helped them with problems, or helped to prevent them drinking, should be thorough and provide detailed insight and knowledge. This approach would allow the authorities to focus on the real reasons why adolescents drank and recognize alcohol as a symptom of something, instead of just a problem to be solved. For example, adolescents could drink because of depression or conflict at home. The participants felt that social and healthcare workers would be aware of the need to focus on the root cause. Some participants described situations when professionals, such as a psychologist, psychiatrist or youth worker, had successfully helped adolescents to tackle problems related to alcohol use.

“Usually the alcohol use is seen as the problem, but the focus should be on the problems that cause the drinking. Often, the use of alcohol is a side effect of a real reason that should be resolved.” (Group 18).

However, some of the participants said that the school nurses had just told them basic facts, such as the harmful consequences of alcohol use. They asked if the adolescents had tasted alcohol, but nothing more. They also pointed out that no one remembered information that teachers read from a book. The adolescents felt that traditional lectures about health hazards were often ineffective, superficial and less helpful than preventive counseling and support. It did not help if teachers shouted and ranted on endlessly about the same negative consequences of alcohol use that they had already heard several times.

“The school nurse always asks about alcohol use, but not in a very profound way.” (Group 2).

“If you tell the school nurse that you have tasted alcohol, then she will ask more about it but she does not provide any further information about it.” (Group 1).

Authorities could successfully offer detailed preventive support for adolescents by arranging lessons where recovered alcoholics could share serious and concrete real-life examples of the consequences of their excessive alcohol use. These examples helped adolescents to think and gave them a greater understanding of the fact that alcohol was a dangerous substance. Many adolescents felt that awareness of the serious consequences would help them to make better decisions about alcohol and motivate them to avoid it. One girl pointed out that showing detailed documentary films was also an effective deterrent.

“It would influence adolescents if a former alcoholic came to school and talked about their experiences, about how terrible it can be if you start drinking too young and the difficulties of becoming sober. It would inspire adolescents to think for themselves.” (Group 15).

“There are documentaries about smoking and drinking which, for example, could show x-ray images of the changes in the brain structure when someone drinks a certain amount. This could show how it affects the body, what happens in the brain and how it affects everyday life and your future.” (Group 12).

## 4. Discussion

This study provides new information about adolescents’ perspectives with regard to responsibilities for their use of alcohol. Overall, adolescents saw alcohol as harmful but tempting. They said that they were developing a sense of responsibility, but also felt that parents had unquestionable responsibility for their children. Based on our findings, we believe that if adults got more involved in adolescents’ alcohol use, and provided ways that they could get anonymous and detailed support, it would encourage and support adolescents to develop their own responsibility and decrease their alcohol use.

The adolescents discussed the benefits, harms and negative consequences of alcohol use thoroughly and did not believe it had a place in their everyday life. They wanted to have good health now and in the future and emphasized the senselessness of drinking. Their belief that they had a responsibility to take care of themselves and their peers’ health was in line with existing research [22]. According to previous studies, adolescents perceived alcohol to be even more harmful than cannabis [40]. However, the reasons our participants gave for using alcohol were also in line with other studies [14,15,16]. These included belonging to a group, regulating and controlling their emotions, having new experiences and excitement. Research has found that some adolescents had not considered the harmful consequences of alcohol use at all [41] and did not feel they were responsible for them [15]. Instead, adolescents said that some adolescents seemed to feel that seeking new experiences could be more important than having a good relationship with their parents. The feedback we received from adolescents underlines the importance of asking them why they drink and using the answers to provide them with the support they need to meet their responsibilities and fulfill their needs.

We found that while adolescents said that they were developing their sense of responsibility, they needed their parents to assume overall responsibility. These findings were in line with previous research that pointed out that parents were primarily responsible for caring for their children. Parents also had the main responsibility for adolescents’ alcohol use, even when they were not physically present when the adolescents were drinking [42]. The adolescents in our study were split on the issue of parents adopting a permissive approach, with some of them supporting clear, even strict, rules and punishments. Previous research has shown that adolescents’ alcohol use was reduced if parents set restrictive rules [43,44] and stopped or discouraged children from drinking [45]. Being punished by parents has been reported to both ease and complicate adolescents’ willingness to take responsibility for their health [22]. Other adolescents in our study described that total prohibition could cause defiance and some adolescents could increase their alcohol use to control these feelings. They felt that punishment was useless, as reported by a previous study [46]. The adolescents described that it was senselessness for parents to allow adolescents to drink without restrictions and this finding was in line with previous research. Permission to drink has been found to increase early alcohol initiation, heavier use and other problem behavior [44,45]. Overall, most of the adolescents felt that tasting alcohol under parental supervision was responsible.

All this contradictory evidence could provide challenges about how to support adolescents meet their responsibilities for alcohol use. Alcohol is generally accepted, and used, in Finland and 53% of adult males and 28% of females use alcohol each week [47]. The 2019 Finnish school health survey reported that more than half of adolescents aged 14–15 said that it was acceptable to drink small amounts of alcohol and one-third did not condemn drunkenness [48]. Although the adolescents thought that most parents had discharged their responsibilities regarding experimenting with alcohol appropriately, they felt that some were negligent and ignorant about adolescents’ alcohol use. Previous research has suggested that parents may have limited capacity to deter adolescents’ drinking by setting rules [44] and neglectful parenting increased drinking among adolescents [46]. However, the views expressed by the adolescents in our study could reflect their parents’ attempts to show that they trusted their children. The parents may have wanted to give adolescents the space to make their own choices and decisions and to practice taking responsibility. Based on our findings, parents may also need support themselves, so that they can meet their responsibilities for adolescents’ alcohol use.

We believe that providing anonymous and easily available services for adolescents would be beneficial and would increase the confidence they have in the adults who are involved in their alcohol use. Anonymity, particularly on the Internet, could make it easier to seek help, especially if adolescents lack the skills, competence or capacity to take responsibility for themselves. The fear of being stigmatized could be a barrier to seeking face-to-face support and thus discourage adolescents from seeking help. Previous research has found promising results for preventive technology-based interventions that aimed to reduce binge drinking among adolescents [49,50]. One used a combination of web-based and text messaging [49] and another used an Internet-based game about alcohol [50].

In addition, we agreed with the views expressed by the adolescents in our study, that hearing the real-life experiences of recovered alcohol users, or experts by experience, would inject a sense of realism and reduce their excitement about alcohol. Based on our interviews, we think that these experts by experience could achieve a genuine rapport with adolescents. They could have an impact on their alcohol use, because their experiences of drinking would teach them not to judge or blame adolescents. By providing real-life examples of how alcohol damage people’s health and futures, they could provide the profound counseling that the adolescents felt they needed.

### 4.1. Strengths and Limitations

The main strength of this study was that we achieved our study aim. This was to describe adolescents’ perceptions and experiences of their responsibilities with regard to their use of alcohol in a trustworthy way. This means that we adhered to the rigorous methods in the consolidated criteria for reporting qualitative research 32-item checklist, particularly in relation to the study design, data analysis and reporting [30].

We described the data collection and analysis process during the design phase of the study to facilitate transferability. Group interviews were chosen for the data collection, as this method has proved to be effective in health research [51]. The semi-structured interview guide enabled us to explore the adolescents’ perceptions and experiences of the focused topic [30]. We asked all the groups the same main interview theme questions, to ensure consistency [39]. Being in a group with friends made the discussions free and easy and encouraged the adolescents to talk and interact with each other. The adolescents listened to their peers’ descriptions carefully, but they also had the courage to disagree in a surprisingly open and mature way. As one girl commented: “It is easy to express your opinion in a small familiar group. I certainly spoke my mind.” (Group 3) To confirm the saturation achieved during the first 15 group interviews, the researcher (M.M) interviewed four more groups. The participation rate was 87/416 (21%) of all ninth-grade students in the selected schools and the amount of data we collected increased the credibility of our findings.

The inductive content data analysis method allowed us to identify a diverse range of perspectives on the topic [39]. To ensure the credibility of the findings, and the interpretation of the data, we discussed grouping the data into categories several times during the analysis. Based on this, we ensured that the identified categories faithfully represented the adolescents’ responses and captured their intended meanings. To enhance the transferability in the reporting, we provided supporting quotations from different participants in every group [30,39]. The adolescents often discussed a number of overlapping themes at one time and we felt it was too complicated to separate these perceptions and experiences from each other in a reliable way and then itemize them. This would not have produced interesting new knowledge or changed the results we have presented.

This study had some limitations. Firstly, the participants were all from a small area of Eastern Finland, so they were a sociodemographically and culturally homogenous group living in a western welfare state. This means that our findings cannot be generalized to all adolescents. Secondly, the results could have been different if we had interviewed institutionalized adolescents living in reformatory schools, because they often have challenging problems with alcohol use and need specific support. The study did not measure, or record, how often the participants drank alcohol, but they had the chance to share their experiences of alcohol use if they wanted to. Our focus was on the adolescents’ subjective perceptions of alcohol use and this could have caused response bias. In addition, participation in this study was voluntary. That could have caused representation bias, as it is possible that the perceptions of our cohort may have differed from those who did not participate. It is possible that some of the adolescents refused to take part because the data was collected during group interviews. Using group interviews also provides other challenges. For example, some participants can crave attention and the need for peer approval can lead to shared views being expressed. This can affect the accuracy of the comments [52], even when there is a relaxed atmosphere. Group interviews can also be dominated by outspoken participants, while shy, quiet ones are less likely to speak up [34]. Although most of our groups were talkative and lively, some participants were quieter and uncertain about how to answer the questions.

### 4.2. Future Directions and Implications

This study provides the basis for further, in-depth, individual qualitative interviews in other contexts to create a broader view of the phenomenon. Research focusing on the perceptions and experiences of responsibilities of adolescents who do not drink at all could provide knowledge that could support, and encourage, adolescents to positively meet their responsibilities for alcohol use. Seeking adolescents’ more detailed views on using so-called experts by experience could widen our understanding of the significant role they could play in preventive counseling.

There is a clear need for more research about the adolescents’ perceptions about how some parents neglected their children when it came to them using alcohol. We need to find out what these perceptions were based on, by using quantitative self-report methods or qualitative interviews with adolescents. This knowledge could help to increase, and guarantee, adolescents’ feelings of security and prevent them from experimenting with alcohol. Authorities could combine interventions that use Internet and text messaging for adolescents, including preventive counseling services. This could promote anonymity for adolescents who want to seek help from school health services [53]. These services could be targeted at large groups of students, irrespective of their possible drinking level [49] and parents could also participate. In order to implement Internet-based interventions, authorities need to increase their involvement in, and adherence to, regional and national eHealth interventions [50,54]. This could make it possible to expand interventions and increase their effectiveness, cost-effectiveness and public health impact [50,55]. These could also be provided on a global basis, including in low-income and middle-income countries [56].

The findings of this study could also be helpful in developing a scale to measure responsibilities in adolescents’ use of alcohol. Comparing different ethnic groups, and parents’ and adolescents’ descriptions, could help to clarify, widen and enrich our knowledge of responsibilities regarding adolescents’ use of alcohol. Since adolescents’ socioeconomic backgrounds influence their perceptions of alcohol [11,13], this knowledge could be useful when it comes to supporting their responsibilities at national and international levels. All this knowledge could increase our understanding of adolescents’ perspectives when future alcohol prevention programs are planned. It could also help us to find the best ways to reduce adolescents’ alcohol use and improve their health.

## 5. Conclusions

This study produced new knowledge on what adolescents felt their responsibilities were with regard to using alcohol. Using this knowledge could help professionals to offer adolescents a stronger basis for their growth and development for a healthy future. Identifying, and focusing on, adolescents’ needs to control their feelings, and to cope with them, could help them to meet their responsibilities with regard to alcohol use. Our overall conclusion was that adolescents’ perceptions and experiences of their responsibilities were reflected in their use of alcohol and their need to belong to peer groups. They also wanted their parents to get involved in how they used alcohol and to care for them. The adolescents were aware of the harmful consequences of alcohol and this was reflected in the fact that they realized that they needed to take care of their health and their peers’ health. This might be one reason of the declined use of alcohol by adolescents in recent decades. In addition, adolescents are developing a sense of responsibility and learning to make healthy responsible decisions and choices on their own. Developing into healthy individuals could raise their awareness of the need to avoid alcohol. It is important that adolescents who need support are helped in a way that meets their own individual needs and that includes understanding why they use alcohol. Based on our findings, parents may also need support to meet their responsibilities when it comes to handling how their adolescents use alcohol.

## Figures and Tables

**Table 1:** Characteristics of the 87 adolescents who took part in the study.

Characteristics		Number of Participants (*N* = 87)	
Gender	Girls	50	(57%)
	Boys	37	(43%)
Age in years	14	7	(8%)
	15	71	(82%)
	16	9	(10%)
Number of family members	2–3	13	(15%)
	4–5	52	(60%)
	6–7	11	(13%)
	8–9	5	(6%)
	10–12	5	(6%)
School area	Rural area	76	(87%)
	Urban area	11	(13%)

**Table 2:** Examples of meaning units and codes in the data analysis process.

Authentic Expressions, Meaning Units Underlined	Code	Subcategory	Category	Main Category
“I am sure that talking about things will help a lot, if you have kept them just to yourself.” (Group 12)	Talking is helpful	Talking about problems	Taking care of their own wellbeing	Adolescents’ developing a sense of responsibility for alcohol use
“You must look for help if you cannot deal with it by yourself.” (Group 16)	Looking for help by themselves	Looking for support and receiving help		
“You can advise friends to decrease their drinking or not let them drink.” (Group 1)	Telling peers not to drink	Providing sensible advice and restrictions	Intervening in peers’ alcohol use	
“My parents will give me a glass of red wine at the Christmas feast. It is quite bad. However, they will not give me the whole bottle.” (Group 15)	Being given parental permission to drink at special events	Tasting alcohol under parental supervision	Permission for alcohol use	Parents’ unquestionable responsibility for adolescents’ alcohol use
“If children know that parents are disinterested, they might drink as much as they like.” (Group 16) “Parents do not want to be responsible for anything extra.” (Group 13)	Parents disinterested Parents do not want to take responsibility	Neglect and abandonment	Parents’ responses to experimenting with alcohol	
“Calling the police is such an unusual response. If someone told me that their parents had done that, I would have said ‘what?’. It would be bad.” (Group 18)	Unusual to call the police	Overprotecting		

**Table 3:** Adolescents’ perceptions and experiences of responsibilities for their use of alcohol.

Subcategory	Category	Main Category
Damages health	Senseless use of alcohol	Harmful, but tempting, use of alcohol
Endangers the future: image and reputation		
Complicates relationships with parents		
Causes additional expense for society		
Sense of belonging to a group	Desired benefits of alcohol use	
A tool to regulate and control feelings		
Having new experiences and excitement		
Making the right decisions	Taking care of their own wellbeing	Adolescents developing a sense of responsibility for alcohol use
Talking about problems		
Looking for support and receiving help		
Abstinence or occasional tasting	Responsible behavior if drinking	
Carefully considered, self-controlled, well behaved drinking		
Ensuring that they drink in safe company		
Providing sensible advice and restrictions	Intervening in peers’ alcohol use	
Refusing to purchase, or offer, alcohol to friends		
Contacting parents or police		
Wanting to protect their friends from getting into trouble		
Sharing knowledge and advice about the consequences of alcohol use	Guidance about responsible decision-making	Parents’ unquestionable responsibility for adolescents’ alcohol use
Sound judgement: teaching the difference between right and wrong		
Total prohibition, strict rules and punishments	Permission for alcohol use	
Tasting alcohol under parental supervision		
Being allowed to drink without supervision		
Daily, appropriate and active supervision	Parents’ responses to experimenting with alcohol	
Neglect and abandonment		
Overprotecting		
Understanding and sympathetic	Anonymity	Important characteristics of support sought from authorities
Non-judgmental		
Reliable obligation of confidentiality		
Focusing on the real reasons for drinking	Detailed insight and knowledge	
Sharing serious and concrete examples from ‘real life’		

## Data Availability

Not applicable.
